# Erection of *Euterranova* n. gen. and *Neoterranova* n. gen. (Nematoda, Anisakidae), with the description of *E*. *dentiduplicata* n. sp. and new records of two other anisakid nematodes from sharks off New Caledonia

**DOI:** 10.1051/parasite/2020053

**Published:** 2020-11-13

**Authors:** František Moravec, Jean-Lou Justine

**Affiliations:** 1 Institute of Parasitology, Biology Centre of the Czech Academy of Sciences Branišovská 31 370 05 České Budějovice Czech Republic; 2 Institut Systématique Évolution Biodiversité (ISYEB), Muséum National d’Histoire Naturelle, CNRS, Sorbonne Université, EPHE, Université des Antilles, rue Cuvier CP 51 75005 Paris France

**Keywords:** Parasitic nematode, Ascaridoidea, New genus, New species, Elasmobranchs, South Pacific Ocean

## Abstract

Helminthological examinations of three species of sharks, *Galeocerdo cuvier*, *Triaenodon obesus* (both Carcharhinidae, Carcharhiniformes) and *Stegostoma fasciatum* (Stegostomatidae, Orectolobiformes) from New Caledonian waters, carried out during 2003–2005, revealed the presence of three species of adult anisakid nematodes referable to *Terranova* Leiper et Atkinson, 1914. However, this genus can no longer be considered valid, because its type species has been designated a *species inquirenda*. Therefore, the present nematodes are assigned to two newly established genera, *Euterranova* n. gen. [type species *E*. *dentiduplicata* n. sp.] and *Neoterranova* n. gen. [type species *N*. *scoliodontis* (Baylis, 1931) n. comb.], based mainly on different labial structures. *Euterranova dentiduplicata* n. sp. from the stomach of *S*. *fasciatum* is mainly characterized by the presence of lips with two rows of denticles. Innominate specimens of *Euterranova* (a female and a third-stage larva) were collected from the digestive tract of *T*. *obesus*. Specimens of *N*. *scoliodontis* were recorded from *G. cuvier*. The two named species are described based on light and scanning electron microscopical examinations. *Neoterranova scoliodontis* has previously been recorded in New Caledonian waters from the same host species. Species previously attributed to *Terranova* are transferred to *Euterranova* (5 species), *Neoterranova* (4 species) or considered *species inquirendae* (10 species). Since *Pseudoterranova* Mozgovoy, 1950 was found to be a *nomen nudum* according to the International Code of Zoological Nomenclature (ICZN), the available name of this genus is *Pseudoterranova* Mozgovoy, 1953. A key to *Porrocaecum*-like nematode genera (*Porrocaecum*, *Pseudoterranova*, *Pulchrascaris*, *Euterranova*, and *Neoterranova*) is provided.

## Abstract (fr)

L’examen helminthologique de trois espèces de requins dans les eaux néo-calédoniennes, *Galeocerdo cuvier*, *Triaenodon obesus* (tous deux Carcharhinidae, Carcharhiniformes) et *Stegostoma fasciatum* (Stegostomatidae, Orectolobiformes), réalisé en 2003–2005, a révélé la présence de trois espèces de nématodes Anisakidae adultes qu’on pourrait référer à *Terranova* Leiper et Atkinson, 1914. Cependant, ce genre ne peut plus être considéré comme valide, car son espèce type a été désignée *species inquirenda*. Par conséquent, les nématodes décrits ici sont attribués à deux genres nouvellement établis, *Euterranova* n. gen. [espèce-type *E. dentiduplicata* n. sp.] et *Neoterranova* n. gen. [espèce type *N. scoliodontis* (Baylis, 1931) n. comb.], principalement sur la base de différentes structures labiales. *Euterranova dentiduplicata* n. sp., de l’estomac de *S. fasciatum,* se caractérise principalement par la présence de lèvres à deux rangées de denticules. Des spécimens non nommés d’*Euterranova* (une femelle et une larve de troisième stade) ont été collectés dans le tube digestif de *T. obesus*. Des spécimens de *N. scoliodontis* ont été trouvés chez *G. cuvier*. Les deux espèces nommées sont décrites sur la base d’examens au microscope photonique et électronique à balayage. *Neoterranova scoliodontis* a déjà été signalé dans les eaux néo-calédoniennes chez la même espèce hôte. Les espèces précédemment attribuées à *Terranova* sont transférées à *Euterranova* (5 espèces), *Neoterranova* (4 espèces) ou considérées comme *species inquirendae* (10 espèces). Puisque *Pseudoterranova* Mozgovoy, 1950 s’est avéré être un *nomen nudum* selon le Code international de nomenclature zoologique (ICZN), le nom disponible de ce genre est *Pseudoterranova* Mozgovoy, 1953. Une clé des genres de nématodes de type *Porrocaecum* (*Porrocaecum*, *Pseudoterranova*, *Pulchrascaris*, *Euterranova* et *Neoterranova*) est fournie.

## Introduction

As stated by Moravec and Justine [29], the taxonomy of anisakid nematodes parasitizing elasmobranchs remains rather confused, mainly because of the inadequate descriptions of many species, and this unsatisfactory situation still exists. This mainly concerns representatives of the controversial genus *Terranova* Leiper et Atkinson, 1914, which contains many species parasitic in elasmobranchs, teleosts, crocodilians, colubrid snakes and, previously, marine mammals (e.g. [1, 14, 32, 35, 41, 43]). Currently, with some original descriptions being either incomplete or inaccurate and some type material either lost or unknown, there is no general consensus on the specific composition of this genus [42]. In addition, the taxonomic status of *Terranova* is questionable and, as indicated by Gibson [14], Deardorff [11] and Bruce and Cannon [10], important interspecific morphological features, such as lip characters, spicule differences or the presence or absence of plectanes, indicate the need for a new generic conception for these species.

The only adult anisakid nematode so far reported from elasmobranchs in New Caledonian waters is *Terranova scoliodontis* (Baylis, 1931), found in the tiger shark *Galeocerdo cuvier* (Péron et Lesueur) (Carcharhinidae) [29]. In the same region, unidentified larvae attributed to *Terranova* have been reported from different species of teleosts [22, 37, 38] and, based on sequence data, some of them were later identified as *Terranova pectinolabiata* Shamsi, Barton et Zhu, 2019 [35] or *Pulchrascaris australis* Shamsi, Barton et Zhu, 2020 [36].

The recent examination of nematodes collected by J.-L. Justine and his students from the sharks *Galeocerdo cuvier*, *Triaenodon obesus* (Rüppel) (both Carcharhinidae, Carcharhiniformes) and *Stegostoma fasciatum* (Hermann) (Stegostomatidae, Orectolobiformes) off New Caledonia during 2003–2005 revealed the presence of three different representatives of *Terranova* (*sensu lato*), one new and one known species, plus one unidentifiable at the species level; these are dealt with below. Since *Terranova* was found to be a *genus inquirendum*, two new genera are proposed to accommodate these species.

## Materials and methods

### Ethics

Big sharks are top predators and thus important for ecology; the sharks used in this study were generally by-catches from other studies or caught by private fishermen and then used for our parasitological survey. All work was conducted in accordance with the laws of the Southern Province of New Caledonia.

## Methods

Sharks were either speared or caught by line. The nematodes were fixed in hot 70% ethanol and preserved in the same liquid. For light microscopical (LM) examination, they were cleared with glycerine. Drawings were made with the aid of a Zeiss microscope drawing attachment. Specimens used for scanning electron microscopical (SEM) examination were postfixed in 1% osmium tetroxide (in phosphate buffer), dehydrated through a graded acetone series, critical-point-dried and sputter-coated with gold; they were examined using a JEOL JSM-7401F scanning electron microscope at an accelerating voltage of 4 kV (GB low mode). All measurements are in micrometres unless otherwise indicated. The fish nomenclature follows FishBase [12].

Parasites other than nematodes, from the sharks listed in this paper, were also collected and studied: they included copepods [9] and trypanorhynch cestodes [6–8]. Compilations of these results have already been published [5, 20, 21].

## Results

Family Anisakidae Railliet et Henry, 1912

### Genus *Euterranova* n. gen.

urn:lsid:zoobank.org:act:D7135A79-71FD-4A2C-8646-CF42C47D8DEC

#### Diagnosis

Ascaridoidea, Anisakidae. Rather large nematodes, widest in midbody. Cuticle slightly transversely striated. Dorsal lip with two double papillae; each subventral lip with one double papilla and lateral amphid. Each lip provided with small, bilobed median elevation armed with two prominent lateral teeth and one row of several median denticles between them; additional row of median denticles may be present somewhat posterior to anterior row. Interlabia absent. Narrow lateral alae present. Deirids well developed, near level of nerve ring. Oesophagus long and narrow. Ventriculus elongate, without ventricular appendix. Intestinal caecum present. Excretory pore between base of subventral lips. Spicules similar, approximately equal in length. Gubernaculum present or absent. Genital papillae numerous. Ventral postcloacal plectane consisting of several transverse plates present. Vulva anterior to midbody. Tail conical; tip without ornamentation. Parasites of elasmobranchs.

Type species: *E*. *dentiduplicata* n. sp.

Other species: *E*. *galeocerdonis* (Thwaite, 1927) n. comb.; *E*. *ginglymostomae* (Olsen, 1952) n. comb.; *E*. *pectinolabiata* (Shamsi, Barton et Zhu, 2019) n. comb.; *E*. *pristis* (Baylis et Daubney, 1922) n. comb.

Etymology: The name *Euterranova* is composed of *Terranova* (the name of a nematode genus) and the prefix *Eu*- (= proper, true). Gender: feminine.

### Remarks

At present, adult anisakid nematodes possessing a cylindrical ventriculus and an intestinal caecum and parasitizing poikilothermic hosts have been assigned to the genera *Terranova* and *Pulchrascaris* Vicente et dos Santos, 1972 [1, 11, 13]. However, the type species of the former genus is a *species inquirenda* and, consequently, *Terranova* should be considered a *genus inquirendum* (see Discussion). Species of the new genus *Euterranova* n. gen. differ from those of *Pulchrascaris* in having well-developed lips, each with an internal median lobe armed with a comb-like dentigerous ridge formed by two prominent lateral teeth and several medial denticles between them (*vs.* lips reduced, without a median lobe; dorsal lip with two large teeth and both subventral lips each with one large tooth) (see also the key at the end the Discussion).

Cephalic structures are generally considered to be very important taxonomic features in the nematode parasites of vertebrates [1, 13] and, in some groups, e.g. in the Cystidicolidae, some genera are based solely on details of the mouth visible only with the use of SEM [27].

### *Euterranova dentiduplicata* n. sp. Figs. 1–3

urn:lsid:zoobank.org:act:DBA2B215-1CDF-4204-BB54-3C8766EA890D

**Figure 1:**
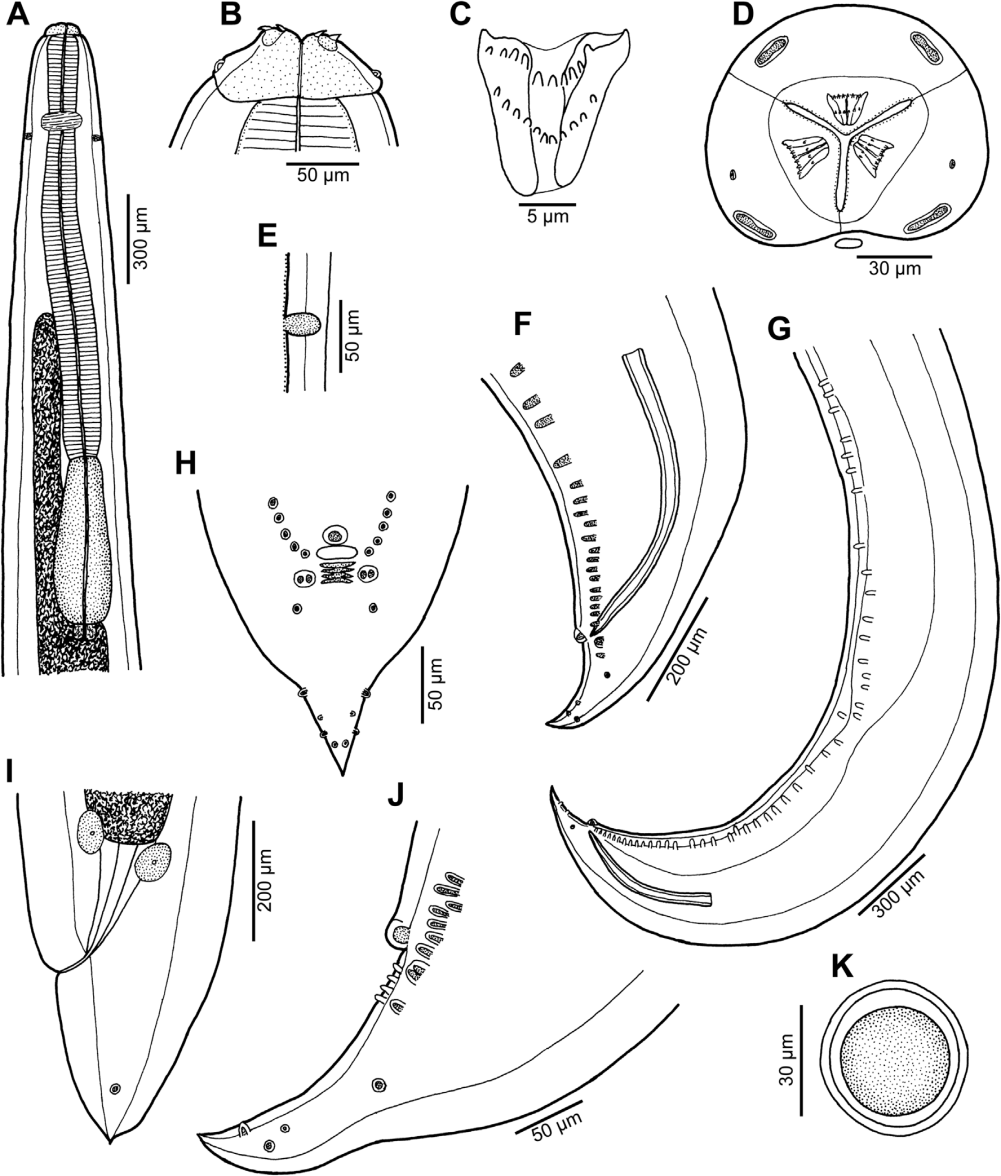
*Euterranova dentiduplicata* n. sp. ex *Stegostoma fasciatum*. (A) Anterior end of male, dorsoventral view; (B) cephalic end of larger male, ventral view; (C) inner surface of median labial elevation armed with teeth in female; (D) cephalic end, apical view; (E) deirid; (F) caudal end of male, lateral view; (G) posterior end of male body, lateral view; (H) male tail, ventral view; (I) tail of female, lateral view; (J) tail of male, lateral view; (K) egg.

Type host: Zebra shark *Stegostoma fasciatum* (Hermann) (Stegostomatidae, Orectolobiformes).

Site of infection: Stomach.

Type locality: Récif Aboré, off Nouméa, New Caledonia (collected 3 May 2005).

Prevalence and intensity: 1 shark infected/1 examined; 19 nematodes.

Details of fish: Parasitological number MNHN JNC1529, total length 208 cm, weight *c*. 30–40 kg. Photographs of the fish deposited into Wikimedia commons (e.g. https://commons.wikimedia.org/wiki/File:Stegostoma_fasciatum_JNC1529_Body.JPG).

Deposition of type specimens: Muséum National d’Histoire Naturelle, Paris (holotype, allotype and 12 paratypes – JNC1529J). Helminthological Collection, Institute of Parasitology, Biology Centre of the Czech Academy of Sciences, České Budějovice, Czech Republic (2 paratypes – Cat. No. N–1245).

Etymology: The specific name of this nematode *dentiduplicata* (= double-indented) is a Latin adjective relating to the characteristic feature of this species, i.e. the presence of two rows of denticles on each lip.

### Description

**Figure 2:**
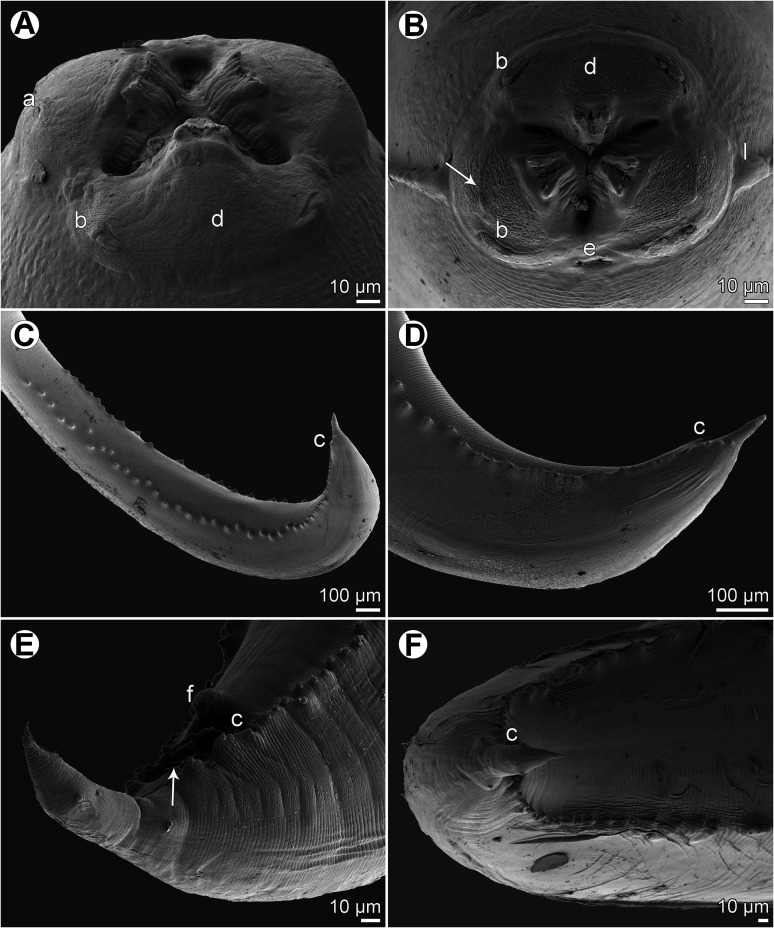
*Euterranova dentiduplicata* n. sp. ex *Stegostoma fasciatum*, scanning electron micrographs of male. (A and B) Cephalic end, dorsal and apical views, respectively (arrow indicates amphid); (C) posterior end of body, ventrolateral view; (D) caudal end, lateral view; (E and F) caudal end of another specimen, sublateral and ventral views, respectively (arrow indicates plectane). (a) Amphid; (b) labial double papilla; (c) cloaca; (d) dorsal lip; (e) excretory pore; (f) median precloacal papilla-like organ.

**Figure 3:**
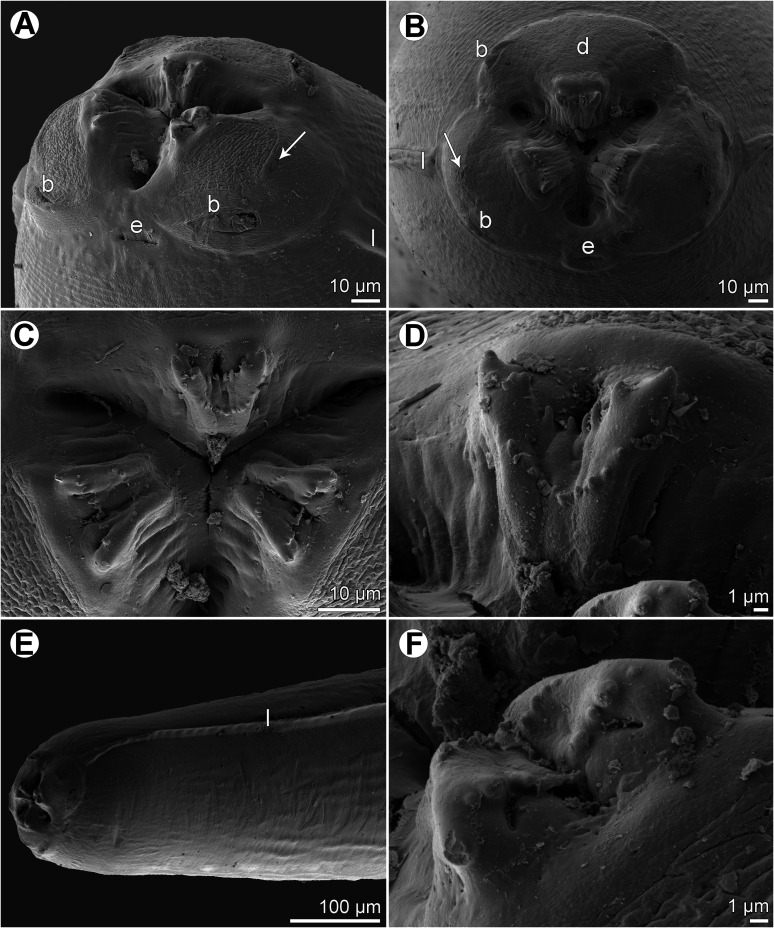
*Euterranova dentiduplicata* n. sp. ex *Stegostoma fasciatum*, scanning electron micrographs. (A and B) Cephalic end of two different males, subventral and apical views, respectively (arrow indicates amphid); (C) detail of median labial elevations in male, apical view; (D) inner side of labial elevation with two rows of teeth in male; (E) anterior end of female, sublateral view; (F) median labial elevation in female, apical view. (b) Labial double papilla; (d) dorsal lip; (e) excretory pore; (l) lateral ala.

*General*: Large, whitish nematodes with thick, transversely striated cuticle (Figs. 2B, 2E, 3A and 3B). Maximum width near middle of body. Lips almost equal in size; inner margins of lips rounded; each lip provided with small, bilobed median elevation armed with 2 prominent lateral teeth and row of 6–10 median denticles between them; additional row of 8–10 median denticles present, being located somewhat posterior to anterior row (Figs. 1B–1D, 2A, 2B, 3A–3D and 3F). Dorsal lip bearing 2 subdorsal double papillae in approximately its basal third; each ventrolateral lip with 1 double subventral papilla and lateral amphid (Figs. 1C, 2A, 2B, 3A and 3B). Interlabia absent. Very narrow lateral alae extending along body present (Figs. 2B, 3A and 3E). Deirids well developed, situated just posterior to level of nerve ring (Figs. 1A and 1E). Oesophagus long, narrow (Fig. 1A). Ventriculus elongate, *c*. 3 times longer than wide, approximately 2.5 times shorter than oesophagus. Caecum long, extending considerably anterior to ventriculus (Fig. 1A). Excretory pore situated ventrally between bases of ventrolateral lips (Figs. 1D, 2B, 3A and 3B). Tail of both sexes conical.

*Male* (6 specimens; measurements of holotype in parentheses): Length of body 19.15–26.58 (25.64) mm; maximum width 707–1,020 (816). Lips 21–51 (39) long. Length of oesophagus 1.48–2.01 (1.82) mm, representing 7–8 (7)% of body length; maximum width 122–204 (163). Nerve ring and deirids 354–422 (422) and 381–666 (462), respectively, from anterior extremity. Ventriculus 517–789 (612) long; maximum width177–218 (204); width/length ratio of ventriculus 1:2.53–3.62 (1:3.00). Intestinal caecum 1.06–1.43 (1.43) mm long, 54–122 (82) wide; length ratio of ventriculus and caecum 1:1.74–2.33 (1:2.33). Posterior end of body curved ventrally. Spicules 530–721 (721) long, representing 2.3–3.0 (2.8)% of body length. Gubernaculum absent. Total of 49–64 (63) pairs of small subventral papillae present, 43–58 (57) being preanals, 1 adanal and 3 (3) postanals; additional 2 pairs of lateral postanals and 1 pair of small lateral phasmids present; first pair of postanal papillae doubled; phasmids situated short distance anterior to level of posterior pair of lateral postanal papillae (Figs. 1F–1H, 1J and 2C–2F). Median preanal papilla-like organ on anterior cloacal lip well developed, fairly large (Figs. 1F–1H, 1J and 2E). Well-developed plectane present posterior to cloacal aperture, being composed of 4 transverse cuticular plates with digitiform lateral extremities (Figs. 1H, 1J and 2E); these ends resemble papillae in lateral view (Fig. 1J). Tail 190–218 (204) long, pointed.

*Female* (5 gravid specimens; measurements of allotype in parentheses. Measurements of 2 non-gravid specimens in brackets): Length of body 31.38–38.09 (37.66) [27.51–28.97] mm; maximum width 1.02–1.36 (1.06) [1.01–1.10] mm. Lips 54–82 (54) [54–68] long. Length of oesophagus 1.99–2.24 (2.03) [1.84–2.01] mm, representing 5–6 (5) [6, 7]% of body length; maximum width 177–218 (204) [136–204]. Nerve ring and deirids 449–476 (462) [435–476] and 476–503 (503) [476–517], respectively, from anterior extremity. Ventriculus 721–816 (816) [694–721] long; maximum width 231–272 (272) [204–218]; width/length ratio of ventriculus 1:2.80–3.30 (1:3.00) [1:3.18–3.53]. Intestinal caecum 1.39–1.61 (1.39) [1.24–1.47] mm long; maximum width 82–136 (177) [95–122]; length ratio of ventriculus and caecum 1:1.70–2.03 (1:1.70) [1:1.72–2.12]. Vulva situated 7.93–10.00 (9.66) [7.75–8.41] mm from anterior extremity, at 23–28 (26) [27–31]% of body length; vagina directed posteriorly from vulva. Eggs in uterus spherical, thin-walled, smooth, 42–48 (42–48) in diameter, with uncleaved content (Fig. 1K). Tail 354–408 (449) [354–435] long, with pair of lateral phasmids near posterior end (Fig. 1I].

### Remarks

This new species is easily distinguishable from other congeners in possessing two (instead of one) transverse rows of denticles on the anterior margin of lips, which is a unique feature within all anisakid nematodes. Of the specimens examined, the second (lower) row of denticles was not clearly visible only in the smallest male.

Bruce and Cannon [10] studied an immature female nematode (16.2 mm long), identified by them as *Terranova* (= *Euterranova*) *ginglymostomae*, collected from the spiral valve of *Stegostoma fasciatum* in Moreton Bay, southern Queensland, Australia. Their specimen was not examined by SEM. Considering the host species and the geographical region, it is highly probable that, in fact, it belonged to *E*. *dentiduplicata* n. sp.

*Euterranova* (as *Terranova*) *ginglymostomae* was described by Olsen [33] from *Ginglymostoma cirratum* (Bonnaterre) (Ginglystomatidae, Orectolobiformes) in the northern West Atlantic (off Florida, USA). Later, based on specimens collected by Johnston and Mawson [19] and identified as *T*. (= *E*.) *galeocerdonis*, it was reported by Bruce and Cannon [10] from *Orectolobus maculatus* (Bonnaterre) (Orectolobidae, Orectolobiformes) off southeastern Queensland, Australia. In contrast to *E*. *dentiduplicata* n. sp., specimens of *E*. *ginglymostomae* are smaller (males and females 17.9–19.1 and 22.3 mm long, respectively, *vs.* 19.1–26.6 and 27.5–38.1 mm, respectively), their lips have only one row (*vs.* two rows) of denticles and the ventral postcloacal plectane consists of 5–6 (*vs.* 4) transverse plates [31].

### *Euterranova* sp. Figs. 4, 5

Host: Whitetip reef shark *Triaenodon obesus* (Rüppel) (Carcharhinidae, Carcharhiniformes).

**Figure 4:**
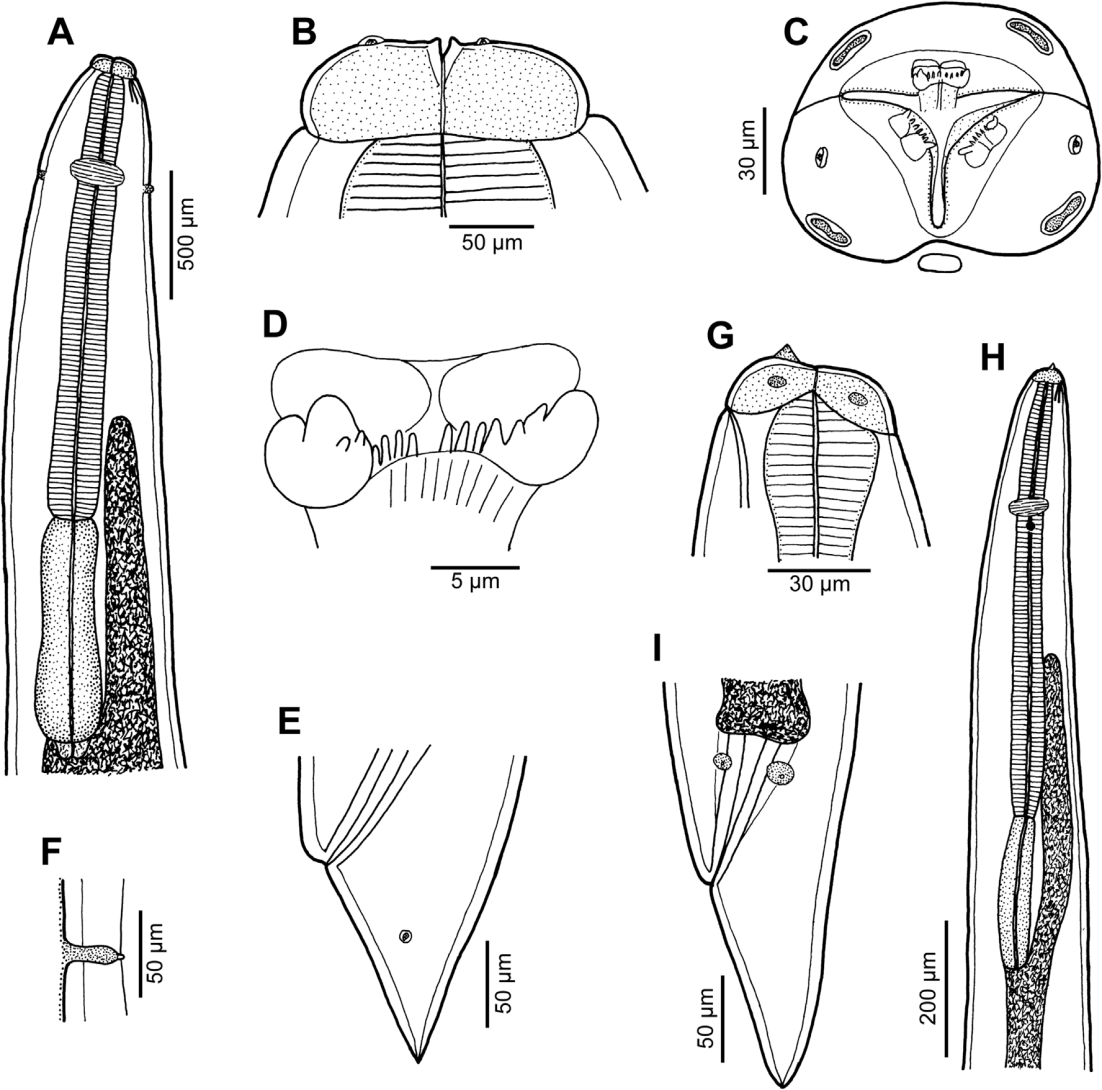
*Euterranova* sp. ex *Triaenodon obesus*. (A–F) Gravid female (A, anterior end, dorsoventral view; B and C, cephalic end, dorsoventral and apical views, respectively; D, inner surface of median labial elevation with row of teeth; E, tail, lateral view; F, deirid). (G–I) Third-stage larva (G, cephalic end, lateral view; H, anterior end of body, sublateral view; I, tail, lateral view).

**Figure 5:**
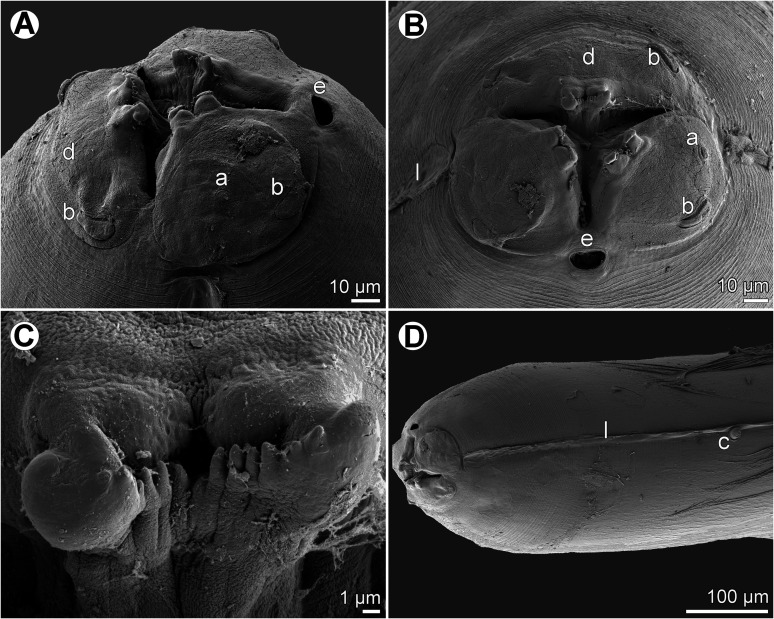
*Euterranova* sp. ex *Triaenodon obesus*, scanning electron micrographs of gravid female. (A and B) Cephalic end, lateral and apical views, respectively; (C) inner side of median labial elevation with teeth; (D) anterior end of body, lateral view. (a) Amphid; (b) labial double papilla; (c) deirid; (d) dorsal lip; (e) excretory pore; (l) lateral ala.

Site of infection: Stomach (adult) and spiral valve (larva).

Locality: Off Nouméa, New Caledonia (collected 4 May 2003 and 10 March 2004).

Prevalence and intensity: 2 sharks infected/2 examined; 1 nematode per shark.

Details of fish: Fish JNC434, length 118 cm, off Nouméa, New Caledonia, 21°55′00″S, 165°45′50″E, 4 May 2003. Fish JNC1054, male, length 110 cm, weight 6.8 kg, off Nouméa, Récif Le Sournois, 10 March 2004.

Deposition of voucher specimens: Institute of Parasitology, Biology Centre of the Czech Academy of Sciences, České Budějovice, Czech Republic (adult mounted on SEM stub – Cat. No. N–1246). Muséum National d’Histoire Naturelle, Paris (larva in vial – JNC434A).

#### Description

*Female* (1 gravid specimen): Large, whitish nematode with finely transversely striated cuticle (Figs. 5A, 5B and 5D). Body length 33.23 mm; maximum width 1.16 mm. Lips almost equal in size, 54 long; inner margins of lips rounded; each lip provided with small, bilobed median elevation armed with 2 prominent lateral teeth and row of about 11 median denticles between them (Figs. 4C, 4D and 5A–5C). Dorsal lip bearing 2 subdorsal double papillae in approximately its basal third; each ventrolateral lip with 1 double subventral papilla and lateral amphid (Figs. 4C, 5A and 5B). Interlabia absent. Very narrow lateral alae extending along body (Figs. 5B and 5D). Deirids well developed, situated just posterior to level of nerve ring (Figs. 4A and 5D), at 517 from anterior extremity. Length of oesophagus 1.95 mm, representing 6% of body length; maximum width 204. Nerve ring 476 from anterior end of body (Fig. 4A). Excretory pore situated ventrally between bases of ventrolateral lips (Figs. 5A and 5B). Ventriculus elongate, 721 long; maximum width 313; width/length ratio 1:2.30. Intestinal caecum 1.36 mm long and 136 wide, extending considerably anterior to ventriculus (Fig. 4A); length ratio of ventriculus and caecum 1:1.89. Vulva situated 8.02 mm from anterior extremity, at 24% of body length; vagina directed posteriorly from vulva. Eggs in uterus spherical, thin-walled, smooth, about 41 in diameter, with uncleaved content. Tail conical, relatively short, with pair of lateral phasmids situated approximately at its mid-length (Fig. 4E); length of tail 313.

*Male*: Not known.

*Third-stage larva* (1 specimen): Body length 5.03 mm; maximum width 190. Cephalic end rounded, with distinct conical larval tooth 9 long and anlagen of developing lips 18 long; excretory pore at level of base of developing lips (Fig. 4G). Length of oesophagus 748; maximum width 45. Nerve ring and deirids 218 and 285, respectively, from anterior extremity. Ventriculus elongate, 231 long and 51 wide; width/length ratio 1:2.30. Intestinal caecum 503 long and 57 wide; length ratio of ventriculus and caecum 1:1.89 (Fig. 4H). Oval genital primordium located at 2.22 mm from anterior extremity, at 44% of body length. Tail conical, pointed, 135 long (Fig. 4I).

#### Remarks

The morphology of the only available adult specimen (female) shows that it belongs to *Euterranova* n. gen. Nevertheless, the structure of lips is different from that in *E*. *dentiduplicata* n. sp. (labial lobes are more prominent and each lip possesses only one transverse row of denticles) and also seems to differ somewhat from *E*. *galeocerdonis* and *E*. *pectinolabiata*, as is apparent from SEM micrographs of these species provided by Tanzola and Sardella [42] and Shamsi et al. [35], respectively. However, in the absence of a male, the specific identification of the available material is impossible.

### Genus *Neoterranova* n. gen.

urn:lsid:zoobank.org:act:31D99978-10C7-470C-A706-2E15E71091AE

#### Diagnosis

Ascaridoidea, Anisakidae. Rather large nematodes with slightly transversely striated cuticle. Dorsal lip with 2 double papillae, each subventral lip with 1 double papilla, 1 single papilla and lateral amphid. Each lip with anterior margin formed into 2 widely separated lobes curved towards median line and median furrow or lobes moderately developed or indistinct, provided with continuous row of even-sized denticles extending along entire inner margin of lips. Interlabia absent. Narrow lateral alae present or absent. Deirids well developed, near nerve ring level. Oesophagus long and narrow. Ventriculus elongate, without ventricular appendix. Intestinal caecum present. Excretory pore between base of subventral lips. Spicules similar, approximately equal in length. Gubernaculum present or absent. Genital papillae numerous. Ventral postcloacal plectane consisting of several transverse plates present. Vulva anterior to midbody. Tail conical; tip without ornamentation. Parasites of sharks and reptiles.

Type species: *N*. *scoliodontis* (Baylis, 1931) n. comb.

Other species: *N*. *caballeroi* (Baruš et Coy Otero, 1966) n. comb.; *N*. *crocodili* (Taylor, 1924) n. comb.; *N*. *lanceolata* (Molin, 1860) n. comb.

Etymology: The name *Neoterranova* is composed of *Terranova* (the name of a nematode genus) and the prefix *Neo*- (= new). Gender: feminine.

#### Remarks

Species of *Neoterranova* n. gen. differ from those of *Pulchrascaris* in having moderately-developed lips, each with a continuous row of even-sized denticles extending along entire, sometimes lobular inner anterior margin (*vs.* lips reduced, without rows of denticles; dorsal lip with two large teeth and both subventral lips each with one large tooth). From those of *Euterranova* n. gen., they differ in having the lips without an inner median lobe armed with a comb-like dentigerous ridge but, instead, with a continuous row of even-sized denticles on each lip (see also the key at the end of the Discussion).

The three above-mentioned species from reptiles, i.e. *N*. *caballeroi*, *N*. *crocodili* and *N*. *lanceolata*, are assigned to this genus tentatively based on the nature of denticles on lips. However, the structure of lips in these species appears to be considerably different from that of the type species (the anterior lobes are moderately developed or rather indistinct) and these species also appear to differ in the structure of postcloacal plectanes, the number and arrangement of male caudal papillae and the presence of a gubernaculum in two of them [41]. Therefore, subsequent detailed studies of these nematodes may indicate the need for a separate genus to accommodate these species.

### *Neoterranova scoliodontis* (Baylis, 1931) n. comb. Fig. 6

Syn.: *Porrocaecum scoliodontis* Baylis, 1931; *Terranova scoliodontis* (Baylis, 1931) Johnston et Mawson, 1945.

**Figure 6:**
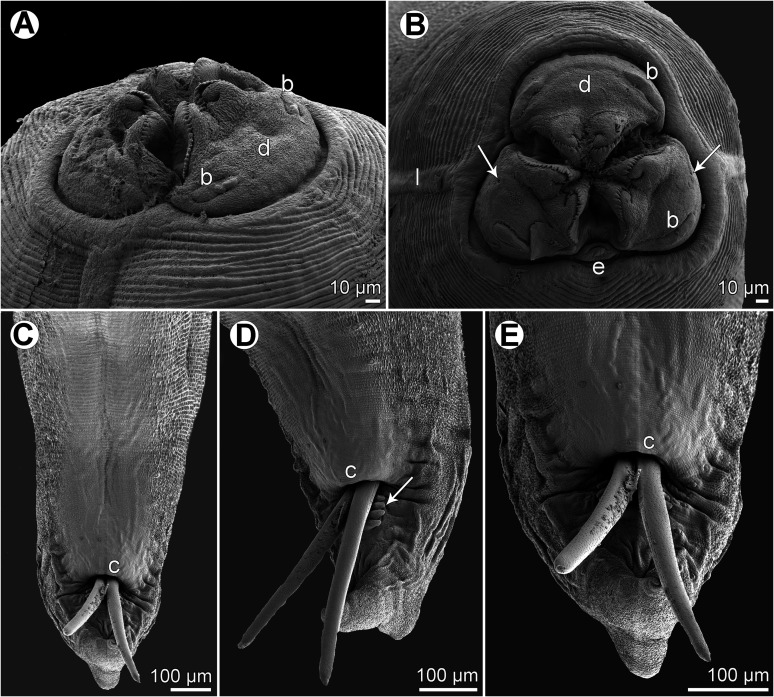
*Neoterranova scoliodontis* (Baylis, 1931) ex *Galeocerdo cuvier*, scanning electron micrographs. (A and B) Cephalic end, sublateral and apical views, respectively (arrows indicate amphids); (C) posterior end of male, ventral view; (D) male tail, subventral view (arrow indicates postcloacal plectane); (E) tail of male (enlarged), ventral view. (b) Labial double papilla; (c) cloaca; (d) dorsal lip; (e) excretory pore; (l) lateral ala.

Host: Tiger shark *Galeocerdo cuvier* (Péron et Lesueur) (Carcharhinidae, Carcharhiniformes).

Site of infection: Stomach and intestine, not in spiral valve.

Locality: Baie de Prony, New Caledonia (collected 20 July 2004).

Detail about fish: Fish JNC1207, female length 341 cm, Baie de Prony, 22°24′S, 166°53′E, 20 July 2004.

Deposition of voucher specimens: Muséum National d’Histoire Naturelle, Paris (MNHN JNC1207).

#### Remarks

A detailed redescription of *E*. *scoliodontis* (as *Terranova*), based on specimens collected from the same host species (*G*. *cuvier*) from off New Caledonia, has already been provided by Moravec and Justine [29]. Since the morphology of the present specimens (two males and three females) is in full agreement with this redescription, we refrain from describing these nematodes once again. The only difference is that Moravec and Justine [29] reported the presence of a poorly developed median preanal papilla in this species, but this was neither observed in the present study (see Figs. 6C–6D) nor previously by Bruce and Cannon [10].

Originally this species was described by Baylis [3] from the carcharhinid shark *Scoliodon* sp. [= probably *Rhizoprionodon acutus* (Rüppel)] [10] off the eastern Australian coast. Gibson and Colin [15] designated it as a junior synonym of the inadequately described *Terranova brevicapitata* (Linton, 1901) from *G*. *cuvier* in the western North Atlantic, but Deardorff [11] resurrected *T*. *scoliodontis*, pointing out that it differs from the former species mainly in the presence of the ventral postcloacal plectane. Based on the LM examination of syntypes, *T*. *scoliodontis* was subsequently redescribed by Bruce and Cannon [10]. Moravec and Justine [29] were the first to study this species using SEM (see above).

## Discussion

The genus *Terranova* was erected by Leiper and Atkinson [25] to accommodate their new species *Terranova antarctica* Leiper et Atkinson, 1914, which was poorly described and based on a single female 32 mm long, collected from the gummy shark *Mustelus antarcticus* (Günther) (Triakidae, Carcharhiniformes) in Bay of Islands, New Zealand [24, 25]. The genus was characterized as follows: “An Ascarid with three large simple lips. No interlabia. Oesophagus simple. Gut with anterior caecal prolongation. No oesophageal appendage.” However, later the type specimen of *T*. *antarctica* was re-examined by Baylis [2], who had assigned it to *Porrocaecum* Railliet et Henry, 1912. Baylis and Daubney [4] considered *Terranova* to be a synonym of *Porrocaecum*, which was followed by some subsequent authors.

Nevertheless, Karokhin [23] proposed the division of *Porrocaecum* into two subgenera based on the presence or absence of interlabia: *Porrocaecum* [type species *P*. *crassum* (Deslongchamps, 1824)] including parasites of birds and *Terranova* [type species *T*. *decipiens* (Krabbe, 1878)] comprising species from elasmobranchs, teleosts, aquatic reptiles and marine mammals. However, since *Terranova* Karokhin, 1946 has a different type species than *Terranova* Leiper et Atkinson, 1914, these names are homonyms according to the International Code of Zoological Nomenclature (ICZN) [16].

Johnston and Mawson [18] resurrected *Terranova* Leiper et Atkinson, 1914 as an independent genus, assigning to it eight other species previously listed in *Porrocaecum*. Of these, Mozgovoy [32] excluded *T*. *kogiae* Johnston et Mawson, 1939, a parasite of pygmy sperm whales, on the basis of the excretory pore allegedly situated at the level of the nerve ring [17], and created a new genus *Pseudoterranova* Mozgovoy, 1953 to accommodate it. However, Gibson [14] proved in type specimens of *T*. *kogiae* that their excretory pore is situated between subventral lips as in other species listed in *Terranova* (*sensu lato*). Mozgovoy [32] listed a total of 13 species in *Terranova* (*s*. *l*.), excluding those described from larvae parasitizing fishes.

Gibson [14], on the basis of some morphological differences, placed the species of *Terranova* (*s*. *l*.) parasitizing mammals in a separate genus *Pseudoterranova* Mozgovoy in Skryabin et al., 1951 [sic], with *Phocanema* Myers, 1959 [type species *P*. *decipiens* (Krabbe, 1878)] as a junior synonym, and this has been followed by subsequent authors (e.g. [26, 28, 44]). However, Mozgovoy [30] listed “*Pseudoterranova* nov. gen.” in his paper of 1950, but this is a *nomen nudum* according to the ICZN, because no other information was provided. In 1951, Mozgovoy [31] published a paper dealing with anisakids of mammals in the then USSR (see [39]), but *Pseudoterranova* is not mentioned in it; on the contrary, he listed *Terranova* to be a valid genus for *T*. *decipiens*. Therefore, the name *Pseudoterranova* accompanied by information on the type species, was first available in the monograph of Mozgovoy (1953) [32], and consequently, this genus should be correctly cited as *Pseudoterranova* Mozgovoy, 1953 (see ICZN, Articles 50 and 21).

Gibson and Colin [14] considered *Terranova*-like species from marine mammals to belong to *Pseudoterranova* (see above) and those having no distinct lips from elasmobranchs and teleosts to *Pulchrascaris* Vicente et dos Santos, 1972 (type species *P*. *caballeroi* Vicente et dos Santos, 1972). The validity of the latter genus was confirmed by Deardorff [11], who redefined it and carried out a detailed review. *Pulchrascaris* has been recognized by subsequent authors (e.g. [10, 36]). The remaining nominal species of *Terranova* (*s*. *l*.) were split by Gibson and Colin [14] into five groups distinguished by the width of the labial prolongations or by their host types, with a sixth group containing *species inquirendae* or *incertae sedis*. Without supporting data, they also synonymized several species, but Deardorff [11] and Bruce and Cannon [10] disagreed with that action and resurrected four species.

Recently Shamsi et al. [35] considered the following nine species of *Terranova* (*s*. *l*.) as valid: *T*. *amoyensis* Fang et Luo, 2006; *T*. *antarctica*; *T*. *brevicapitata* (Linton, 1901); *T*. *edcaballeroi* Díaz-Ungría, 1970; *T*. *galeocerdonis* (Thwaite, 1927); *T*. *pectinolabiata*; *T*. *pristis* (Baylis et Daubney, 1922); and *T*. *scoliodontis* (Baylis, 1931). However, they omitted three congeners parasitizing reptilian hosts in addition to other species of this genus which are parasitic in elasmobranchs and teleosts, such as *T*. *cephaloscyllii* (Yamaguti, 1941) and *T*. *serrata* (Drasche, 1884).

According to Bruce and Cannon [10], there are important interspecific morphological features among *Terranova* spp., such as the presence/absence of lateral alae, plectanes or a gubernaculum, and especially labial characters, which might be used for splitting the genus. In our opinion, the most important differences occur, as in many other nematode groups, at the cephalic end, i.e. the lips and their equipment with teeth. Unfortunately, some significant morphological details, e.g. labial structures, are not readily visible in these fairly large nematodes under the LM and, consequently, these were either inadequately described or undescribed in the great majority of *Terranova* species. To date, only a few *Terranova*-like species from poikilothermic hosts have been studied using the SEM [10, 11, 29, 35, 36, 42].

Since *T*. *antarctica*, the type species of *Terranova*, is known only from a single female, and the majority of taxonomically important morphological features in *Terranova* spp. are found in the male, Bruce and Cannon [10] designated this species as a *nomen dubium* or *species inquirenda*, because it cannot be positively identified. However, a genus is objectively determined by its type species (ICZN, Article 61); if *T*. *antarctica* is a *species inquirenda*, then the respective genus becomes a *genus inquirendum*. Consequently, until *T*. *antarctica* [*species inquirenda*] is redescribed from a newly collected topotypical material or molecular data can be extracted from the type specimen, *Terranova* cannot be considered a valid genus.

Although the type specimen of *T*. *antarctica* is still available at the Natural History Museum in London, its possible re-examination with the use of LM would be useless. Morphological details of its mouth require to be studied under the SEM, which can hardly be carried out on the sole type specimen without the risk of its destruction and with uncertain results. Since the original description of *T*. *antarctica*, no adult specimens of this species have been reported. Larvae designated as *Phocanema antarctica* were found in fish by Reimer [34], but this identification is doubtful.

Consequently, we propose two new genera, *Euterranova* n. gen. and *Neoterranova* n. gen., to accommodate some species previously listed in *Terranova* (*s*. *l*.) from poikilothermic hosts. Since these are based mainly on labial characters, only the species in which these features are clearly described are included; all other species of *Terranova* (*s*. *l*.) are considered to be *species inquirendae* and their generic appurtenance can only be elucidated by subsequent studies. These are: *T*. *amoyensis*, *T*. *antarctica*, *T*. *cephaloscyllii*, *T*. *circularis* (Linstow, 1907), *T*. *edcaballeroi* Díaz-Ungría, 1970, *T*. *nidifex* (Linton, 1901), *T*. *petrovi* Mozgovoy, 1950, *T*. *quadrata* (Linstow, 1904), *T*. *serrata* and *T*. *trichiuri* (Chandler, 1935).

The authors are aware that a molecular analysis is needed to confirm the present results, which will be a matter of future studies.

*Terranova* (*s*. *l*.), previously considered a junior synonym of *Porrocaecum* [4], was resurrected as a valid genus as early as 75 years ago [18], which has been followed by the great majority of subsequent authors (see above). Any similarity of representatives of these two genera is based solely on the presence of an intestinal caecum, but otherwise they are unrelated and belong to different ascaridoid families [1, 25]. Nevertheless, until recently, ascaridoid species attributed to *Porrocaecum* have sometimes been reported as parasites of elasmobranchs and teleost fishes. For example, Sood [40], in his comprehensive monograph devoted to fish nematodes from South Asia, has reported 17 nominal species (all adults) of *Porrocaecum*, including 14 species parasitic in teleosts and three, *P*. *galeocerdonis* Thwaite, 1927, *P*. *bengalensis* Lakshmi et al., 1986 and *P*. *tigrini* Lakshmi, 1992, from the same host species, the tiger shark *Galeocerdo tigrinum* (= *G*. *cuvieri*); except for *P*. *galeocerdonis* (= *Euterranova galeocerdonis*), all the 16 above-mentioned species from India are poorly described and illustrated, and should be considered *species dubiae* and *incertae sedis*. Nevertheless, that is why *Porrocaecum* is included in the following key to some ascaridoid genera.

### Key to the genera of adult *Porrocaecum*-like ascaridoid nematodes:

1 Ascarididae. Interlabia present. Excretory pore approximately at level of nerve ring. Parasites of birds …………………………………………………………..………………... ***Porrocaecum***

Anisakidae. Interlabia absent. Excretory pore located between base of subventral lips. Parasites of poikilothermic hosts or marine mammals ……………………………………………………………………………………..…… 2

2 Glandular left filament of excretory system expands further anteriorly than in species from poikilotherms, i.e. gland broad (25–31% of body diameter) in transverse section at approximately middle of oesophagus. Lips have higher profile, are stout, and have more protruded anterior lobes. Parasites of marine mammals ……………………………………………………………………….. ***Pseudoterranova***

Glandular left filament of excretory system extends less anteriorly than in species from mammals, i.e. gland quite narrow (5–11% of body diameter) in transverse section at approximately middle of oesophagus. Lips very stout with very low profile; their anterior lobes very shallow or absent. Parasites of poikilothermic hosts …………………….…. 3

3 Lips without dentigerous ridges. Dorsal lip with two large triangular teeth; subventral lips each with single large triangular tooth. Parasites of sharks ……………… ***Pulchrascaris***

Lips with dentigerous ridges ………………………………………………………..…… 4

4 Lips with narrow, comb-like dentigerous ridges formed by two prominent lateral teeth and several medial denticles between them. Parasites of sharks …….…. ***Euterranova*** n. gen.

Lips with broad dentigerous ridges formed by rows of even-sized denticles; prominent teeth absent. Parasites of elasmobranchs, teleosts, crocodilians and snakes ............................................................................................................ ***Neoterranova*** n. gen.

## Conflict of interest

The Editor-in-Chief of Parasite is one of the authors of this manuscript. COPE (Committee on Publication Ethics, http://publicationethics.org), to which Parasite adheres, advises special treatment in these cases. In this case, the peer-review process was handled by an Invited Editor, Jérôme Depaquit.
